# ^1^H NMR Signals from Urine Excreted Protein Are a Source of Bias in Probabilistic Quotient Normalization

**DOI:** 10.1021/acs.analchem.2c00466

**Published:** 2022-05-03

**Authors:** Gonçalo D. S. Correia, Panteleimon G. Takis, Caroline J. Sands, Anna M. Kowalka, Tricia Tan, Lance Turtle, Antonia Ho, Malcolm G. Semple, Peter J. M. Openshaw, J. Kenneth Baillie, Zoltán Takáts, Matthew R. Lewis

**Affiliations:** † Section of Bioanalytical Chemistry, Division of Systems Medicine, Department of Metabolism, Digestion and Reproduction, Imperial College London, South Kensington Campus, London SW7 2AZ, United Kingdom; # National Phenome Centre, Imperial College London, Hammersmith Campus, IRDB Building, London W12 0NN, United Kingdom; ¶ Division of Diabetes, Endocrinology and Metabolism, Department of Metabolism, Digestion and Reproduction, Imperial College London, Du Cane Road, London W12 0NN, United Kingdom; § Clinical Biochemistry, Blood Sciences, North West London Pathology, Charing Cross Hospital, London W6 8RF, United Kingdom; □ NIHR Health Protection Research Unit in Emerging and Zoonotic Infections, Institute of Infection and Global Health, University of Liverpool, Liverpool L69 7BE, United Kingdom; ■ MRC-University of Glasgow Centre for Virus Research, University of Glasgow, Glasgow G61 1QH, United Kingdom; ○ NIHR Health Protection Research Unit in Emerging and Zoonotic Infections, Institute of Infection, Veterinary and Ecological Sciences, University of Liverpool, Liverpool L69 7BE, United Kingdom; ● Respiratory Medicine, Alder Hey Children’s Hospital, Liverpool L12 2AP, United Kingdom; △ Faculty of Medicine, National Heart and Lung Institute, Imperial College London, London SW3 6LY, United Kingdom; ▲ Roslin Institute, University of Edinburgh, Edinburgh EH25 9RG, United Kingdom

## Abstract

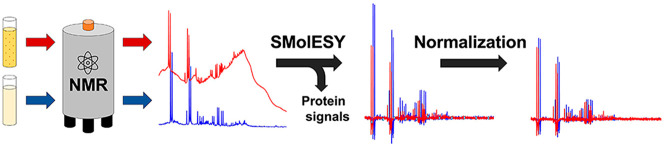

Normalization to account for variation in urinary dilution is crucial for interpretation of urine metabolic profiles. Probabilistic quotient normalization (PQN) is used routinely in metabolomics but is sensitive to systematic variation shared across a large proportion of the spectral profile (>50%). Where ^1^H nuclear magnetic resonance (NMR) spectroscopy is employed, the presence of urinary protein can elevate the spectral baseline and substantially impact the resulting profile. Using ^1^H NMR profile measurements of spot urine samples collected from hospitalized COVID-19 patients in the ISARIC 4C study, we determined that PQN coefficients are significantly correlated with observed protein levels (*r*^2^ = 0.423, *p* < 2.2 × 10^–16^). This correlation was significantly reduced (*r*^2^ = 0.163, *p* < 2.2 × 10^–16^) when using a computational method for suppression of macromolecular signals known as small molecule enhancement spectroscopy (SMolESY) for proteinic baseline removal prior to PQN. These results highlight proteinuria as a common yet overlooked source of bias in ^1^H NMR metabolic profiling studies which can be effectively mitigated using SMolESY or other macromolecular signal suppression methods before estimation of normalization coefficients.

Urine is a complex chemical mixture which contains metabolic end-products from host and associated microbiota, xenobiotics, and dietary compounds^1^ in highly variable concentrations.^2^ Urinalysis is routinely used in the clinic for noninvasive diagnosis of local conditions of urinary tract pathology and infection (e.g., via measurement of leukocytes and nitrite), systemic metabolic disease (e.g., diabetes via glucose), and to assess environmental and nutritional exposure. The ease of sample collection and diagnostic potential has made urine a focal point for biofluid-based metabolomics studies.^3^^1^H nuclear magnetic resonance (NMR) spectroscopy emerged early in the evolution of the field^4^ as a suitable platform for such investigations owing to its broad linear dynamic range, excellent reproducibility, high throughput, and quantitative accuracy.^5−7^

Urine exhibits strong intraday and interindividual variation in dilution owing to factors such as hydration status, kidney function, diet, medication, and voiding interval. This poses a fundamental challenge in urinalysis, especially of spot urine samples, that must be accounted for when accurately making or comparing chemical measurements^8^ in both clinical applications and metabolic profiling studies.^9^ Measurements of urinary creatinine, osmolality, specific gravity, or volume (in 24h collections) are routinely used in clinical biochemistry to account for variable dilution (e.g., albumin-to-creatinine ratio) and allow comparison to normal reference ranges.^10^ However, in metabolomics studies, the use of profiling technologies such as ^1^H NMR allows for the estimation of more robust normalization coefficients based on a broader view of the urinary metabolome. Probabilistic quotient normalization (PQN)^11^ leverages the data captured in the metabolic profile as a whole and is the “gold standard” for computational normalization of ^1^H NMR spectra in urinalysis studies. PQN uses the complete set of profile measurements to estimate a normalization coefficient for each sample that is representative of its dilution factor relative to a predefined reference (usually a median spectrum). Compared to other normalization methods (e.g., total area normalization), PQN provides robustness against bias from few signals whose intensity dominates the total profile integral. However, PQN is not suitable if a large proportion of variables (50% or more) covary systematically with factors other than the sample dilution.^11^

The presence of urinary proteins can exert a broad effect on ^1^H NMR-based metabolic profiles by elevating the spectral baseline and contributing broad signals in a concentration-dependent manner (Figure 1a).^12^ Because a large proportion of signals across the profile are affected by the presence of substantial proteinuria, the fitness of PQN normalization for samples representative of many phenotypes may be challenged. Proteinuria is frequently encountered in chronic kidney disease,^13^ diabetes^14^ and obesity,^15^ and increases with age.^16^ Surprisingly, a thorough literature search did not reveal any critical assessment of the impact of proteinuria in the estimation of normalization coefficients. The ongoing Covid-19 pandemic and organized efforts to understand its underlying pathophysiological effects have provided both a need and the opportunity to evaluate this relationship, as proteinuria is prevalent in COVID-19 patients, independent of other comorbidities, and associated with disease severity and patient survival.^17,18^

**Figure 1:**
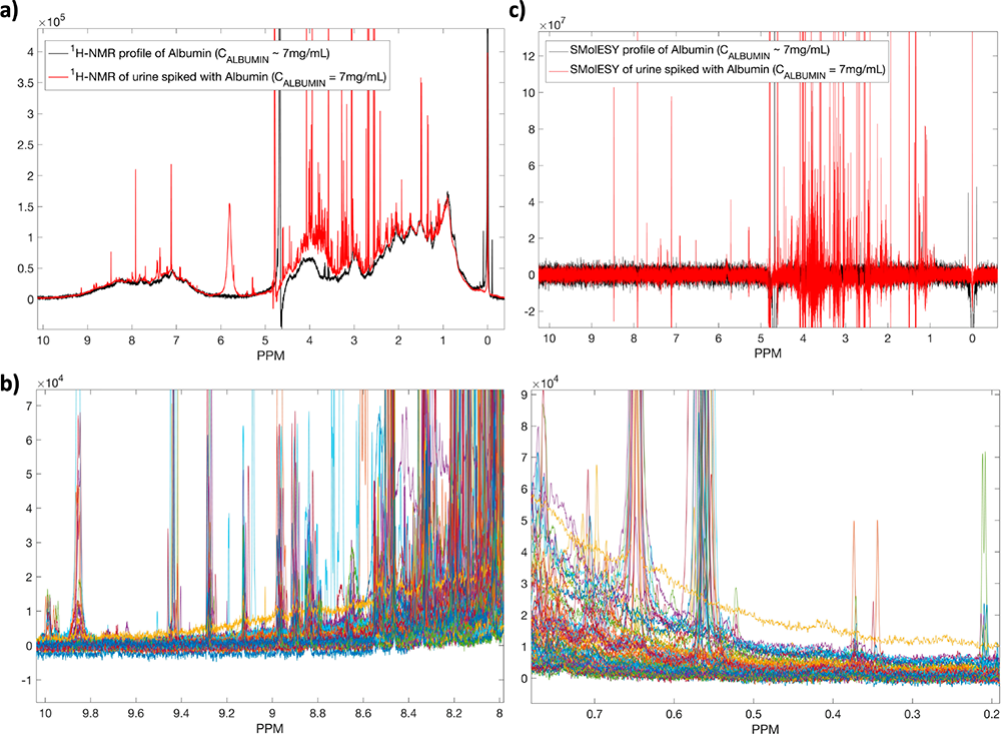
(a) The ^1^H NMR spectrum of albumin (black line) compared to a real urine sample containing the same amount of albumin (∼7 mg/mL). (b) Eighty-five urine ^1^H NMR profiles from COVID-19 patients, focusing on the backbone −NH (left panel) and parts of methyl proteinic protons (right panel), showcasing the spectral baseline effect from the presence of proteinuria. (c) SMolESY application on the ^1^H NMR spectrum of albumin (∼7 mg/mL) and a real urine sample with the same amount of albumin. In both cases SMolESY succeeds in broad signals suppression as well as baseline homogenization, allowing the enhancement of sharp signals from small molecules (i.e., metabolites).

We used an ^1^H NMR-based metabolic profiling approach to analyze urine samples from patients (*n* = 1022 spot urine samples from 711 patients) admitted to hospital with COVID-19, collected by the International Severe Acute Respiratory and Emerging Infections Consortium (ISARIC) following the WHO Clinical Characterization Protocol UK (CCP-UK). Further NMR experimental details are reported in the Supporting Information. Figure 1b shows several urine ^1^H NMR profiles (*n* = 85) from COVID-19 patients, focusing on the backbone −NH and parts of methyl proteinic protons, clearly illustrating the effects of proteinuria on the small molecule profiles observed. The impact of macromolecular signals on the small molecule profile may be reduced when employing additional NMR experiments beyond the routine one-dimensional general profile experiment (i.e., transverse relaxation (*T*_2_) spectral editing experiments such as spin–echo pulse sequences^19^). However, the need for these additional experiments may not be anticipated at the outset of a urine profiling study, and their use both increases the experimental cost and decreases analysis throughput. We recently introduced small molecule enhancement spectroscopy (SMolESY)^12^ as a computational alternative for removal of macromolecule-derived signals directly from routine one-dimensional NMR spectral profiles without the need of extra experiments, enabling the more specific and direct analysis of small molecule analytes (Figures 1c, S1, and S2). The approach can also be effectively reversed, removing the sharp small molecule-derived signals and providing an enhanced protein baseline for the purposes of urinary protein quantification (Figure 2a, S3).^20^ Integration of the proteinic methyl group-containing spectral region between 0.2 and 0.5 ppm (Figure 2a) accurately represents the total amount of urinary protein as validated by comparison with turbidimetric measurement (Figure 2b). In the present study, we compared the PQN coefficients obtained from the standard one-dimensional (1D) ^1^H NMR spectra and their SMolESY processed counterparts (Figure 3). Although there is a good correlation between both measures (Pearson’s ρ = 0.89), there is also a visible trend in the deviations from the regression line associated with high levels of total protein quantified in each sample, confirming that proteinuria does influence the estimation of PQN coefficients. Urinary protein excretion alone explains approximately 42.3% (*r*^2^ = 0.423) of the variance in PQN coefficients estimated from the 1D ^1^H NMR spectra without macromolecular baseline removal (Figure 4a). This trend is greatly reduced (*r*^2^ = 0.163) when the macromolecular signature is removed via SMolESY prior to PQN (Figure 4b), closer to the observed association between urinary creatinine and protein concentration (*r*^2^ = 0.063, Figure S5).

**Figure 2:**
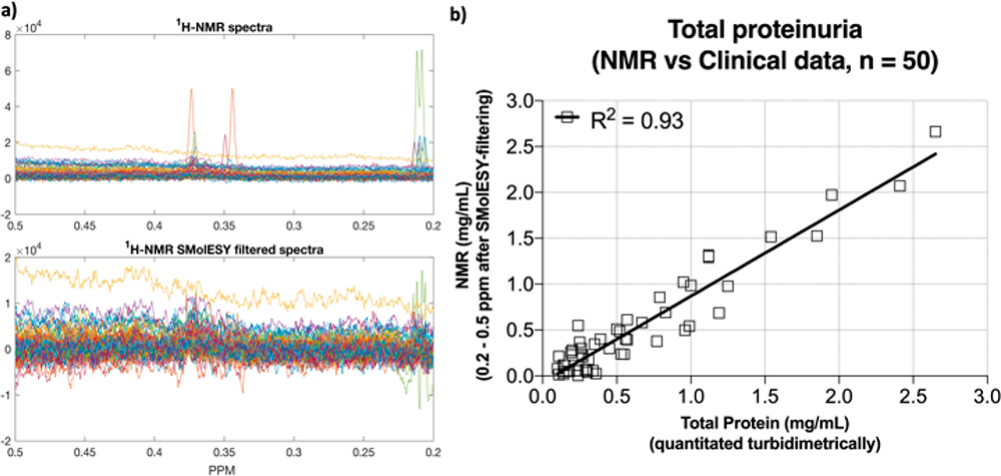
(a) Fifty ^1^H NMR spectra from the COVID-19 cohort focusing on the proteinic methyl group-containing spectral region between 0.2 and 0.5 ppm (upper panel). After processed SMolESY filtering, the resulting spectra (bottom panel) are free from small metabolites sharp signals (or the remaining signals contain the same negative/positive part with almost zero integral) and their integration provides an estimate of the total protein. (b) The absolute quantification of total urinary protein via NMR highly correlates to the measured protein concentration by clinical methods (for both NMR and clinical methods see Supporting Information).

**Figure 3:**
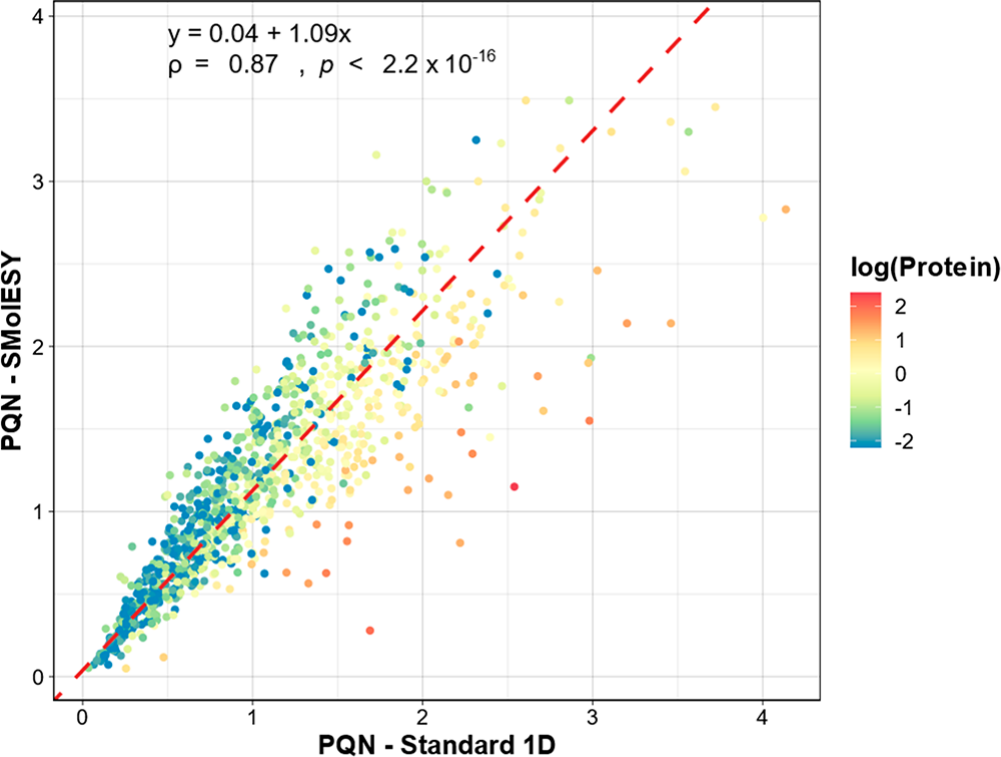
Agreement between PQN coefficients estimated from the standard ^1^H NMR spectra and the corresponding SMolESY processed data. The linear regression trendline (dashed red line) was estimated with the orthogonal least-squares Passing–Bablok method. The regression coefficients, Pearson correlation coefficient (ρ) and the *p*-value from the two-sided correlation significance test are shown in the top left corner. Data points are colored by the natural logarithm of the estimated protein concentration (mg/mL).

**Figure 4:**
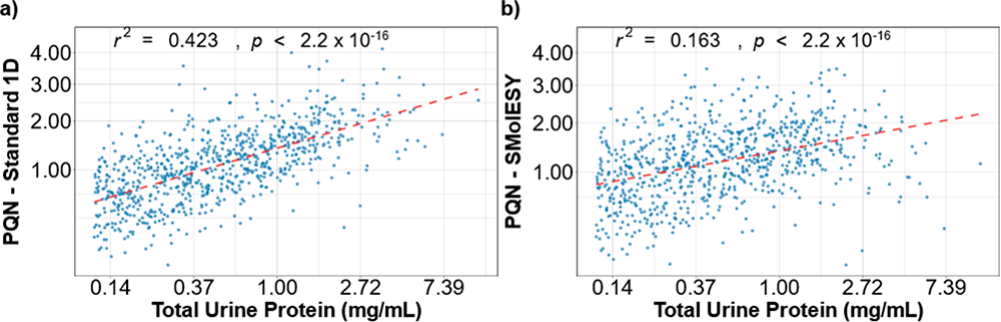
PQN coefficient variance explained (*r*^2^, estimated from the linear regression models plotted in red) by protein concentration, in the (a) standard and (b) SMolESY processed NMR spectra. Protein values equal or below the limit of detection (LOD) = 0.11 mg/mL were excluded for this analysis (final *n* = 810). PQN coefficients were square root transformed, and urine protein measurements were log-transformed.

A comparison between the PQN coefficients and creatinine concentrations is shown in Figure 5. While creatinine concentration can be affected by multiple biological factors, PQN coefficients should still correlate with creatinine levels in spot urine samples, as observed in Figure 5a,b. Although the Pearson correlation between PQN coefficients and creatinine concentration is only marginally improved in the SMolESY data set (ρ = 0.71 vs ρ = 0.69, respectively) the residuals from the linear regression models (dashed red lines in Figure 5a,b) have a strong association with total urine protein (*r*^2^ = 0.43, Figure 5c) in the original spectra, a trend which is reduced in SMolESY data (*r*^2^ = 0.1, Figure 5d).

**Figure 5:**
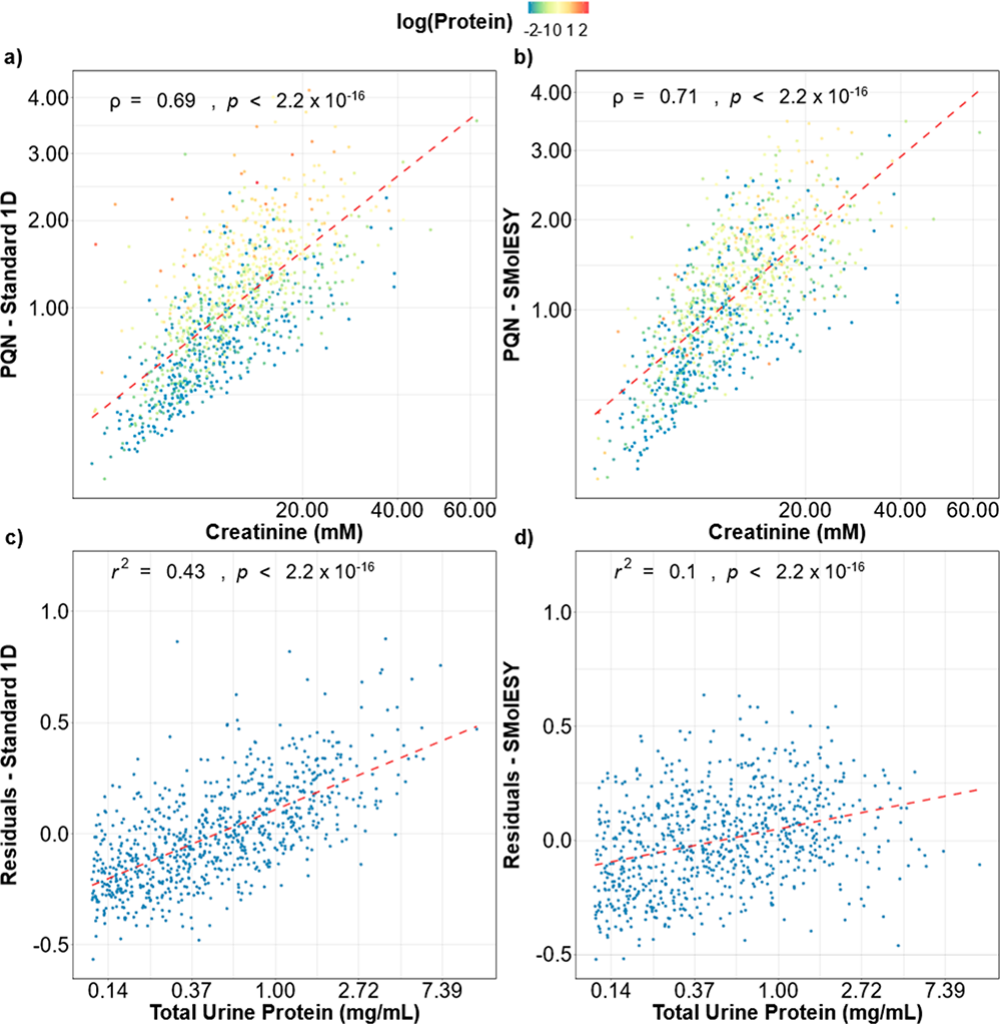
Correlation between PQN coefficients estimated from (a) the standard ^1^H NMR or (b) SMolESY processed spectra and creatinine. Pearson correlation coefficient and *p*-value from the two-sided correlation significance test are shown in each figure. Data points are colored by the natural logarithm of the estimated protein concentration. PQN coefficients and creatinine measurements were square root transformed. (c,d) The residuals from the OLS regression trendlines (dashed red lines in panels a and b) and their residual association with total urine protein (log-transformed). Protein values equal or below the LOD = 0.11 mg/mL were excluded in panels c and d (final *n* = 810).

Figure 6 shows the principal component analysis (PCA) scores plots obtained when the SMolESY processed data set is normalized using PQN coefficients estimated from the standard 1D NMR spectra (Figure 6a) or from SMolESY data (Figure 6b). Despite the similarity in major trends in both plots, clustering based on protein concentration is more marked when spectra are normalized with the standard PQN procedure (Figure 6a).

**Figure 6:**
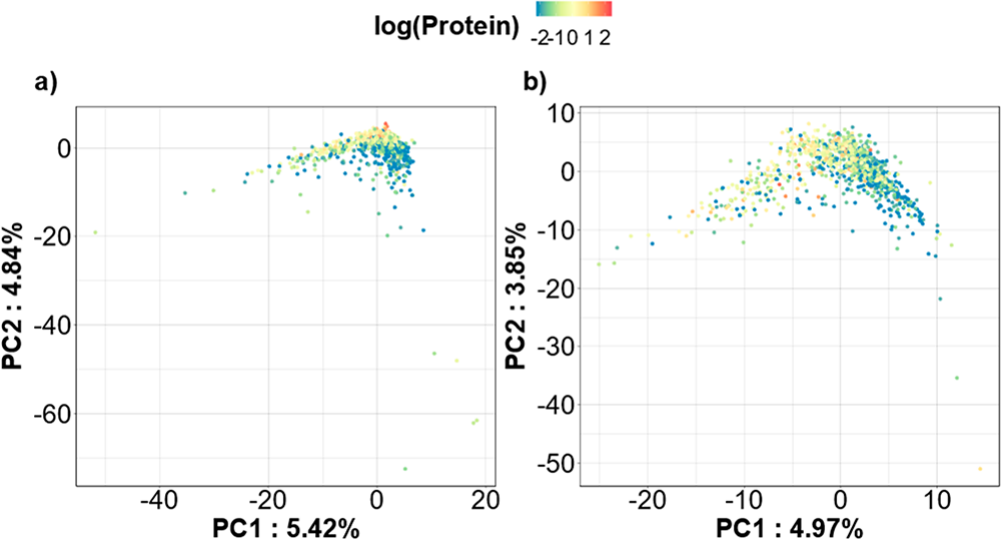
PCA scores plots for the SMolESY processed data set normalized with the PQN coefficients estimated from the (a) standard 1D and (b) SMolESY processed NMR spectra. NMR data was unit-variance scaled. Protein values were log-transformed and values equal or below LOD were imputed by replacement with the LOD value = 0.11 mg/mL.

Normalization procedures are crucial to correctly interpret urinary metabolic profiles. Here, we show that protein signals can confound probabilistic quotient normalization, and it is reasonable to assume this could also happen with other computational normalization methods. We recommend the removal of protein baseline signals prior to estimation of normalization coefficients. This can be performed experimentally^21^ or with computational methods.^22,23^ However, we advocate the use of SMolESY, because of its ease of application to ^1^H NMR spectra (including retrospective application where proteinuria is observed after the fact), being fast and highly effective method for removing broad baseline signals from protein and improving the estimation of normalization coefficients. Our observations and proposed methodology are of high importance for the accurate normalization of urine biofluid ^1^H NMR spectra, especially in the context of studies on diseases and phenotypes where proteinuria is likely to be present (e.g., diabetes, chronic kidney disease, pregnancy, infection, or protein rich diet).^24^

## Supporting Information Available

Urine samples collection details; NMR experiments/apparatus; NMR samples preparation SOPs; SMolESY data preparation; urinary creatinine quantification by ^1^H NMR; urinary total protein quantification by clinical methods and ^1^H NMR/SMolESY; statistical analyses details; PQN coefficients comparison with urinary creatinine concentration and osmolality measurements by clinical methods; correlation between urinary creatinine concentration and protein excretion; comparison between CPMG and SMolESY estimated PQN coefficients in human plasma heparin ^1^H NMR spectra; supporting references (PDF)

## Notes

This work was done on behalf of and used human samples collected by the ISARIC4C consortium. The full membership list can be found in the following link: https://isaric4c.net/about/authors/
