# Supplementary material for: 1H NMR Signals from Urine Excreted Protein Are a Source of Bias in Probabilistic Quotient Normalization
Source: Anal Chem. 2022 May 3;94(19):6919–23. doi: 10.1021/acs.analchem.2c00466 (PMC9118196; doi:10.1021/acs.analchem.2c00466)
Supplement: Supplementary file 1 — ac2c00466_si_001.pdf [file ac2c00466_si_001.pdf]

SUPPORTING INFORMATION

^{1}H NMR signals from urine excreted protein are a source of bias in probabilistic quotient normalization.

Gonçalo D. S. Correia,[1,2,‡,*] Panteleimon G. Takis,[1,2,‡,*] Caroline J. Sands,[1,2] Anna M. Kowalka,[3,4] Tricia Tan,[3,4] Lance Turtle, [5] Antonia Ho, [6] Malcolm G. Semple, [7,8] Peter J. M. Openshaw, [9] J. Kenneth Baillie, [10] Zoltán Takáts,[1,2] and Matthew R. Lewis[1,2]

[1]Section of Bioanalytical Chemistry, Division of Systems Medicine, Department of Metabolism, Digestion and Reproduction, Imperial College London, South Kensington Campus, London, SW7 2AZ, UK

[2]National Phenome Centre, Imperial College London, Hammer-smith Campus, IRDB Building, London, W12 0NN, UK

[3]Division of Diabetes, Endocrinology and Metabolism, Department of Metabolism, Digestion and Reproduction, Imperial College London, Du Cane Road, London, W12 0NN, UK

[4]Clinical Biochemistry, Blood Sciences, North West London Pathology, Charing Cross Hospital, London, W6 8RF, UK

[5]NIHR Health Protection Research Unit in Emerging and Zoonotic Infections, Institute of Infection and Global Health, University of Liverpool, Liverpool, UK

[6]MRC-University of Glasgow Centre for Virus Research, University of Glasgow, Glasgow, UK

[7]NIHR Health Protection Research Unit in Emerging and Zoonotic Infections, Institute of Infection, Veterinary and Ecological Sciences, University of Liverpool, Liverpool, UK

[8]Respiratory Medicine, Alder Hey Children's Hospital, Liverpool L12 2AP, UK

[9]Faculty of Medicine, National Heart and Lung Institute, Imperial College London, London, UK

[10]Roslin Institute, University of Edinburgh, Edinburgh, UK

‡These authors contributed equally.

This work was done on behalf of and used human samples collected by the ISARIC4C consortium. Full membership list can be found in the following link: https://isaric4c.net/about/authors/

Corresponding Authors

*Section of Bioanalytical Chemistry, Division of Systems Medicine, Department of Metabolism, Digestion and Reproduction
Imperial College London, South Kensington Campus, London, SW7 2AZ, UK.
National Phenome Centre, Imperial College London, Hammersmith Campus, IRDB Building, London, W12 0NN, UK.
Email: gd2212@imperial.ac.uk
Email: p.takis@imperial.ac.uk

Table of Contents

Urine Samples collection 3
NMR samples preparation and spectra acquisition 3
SMolESY data preparation – Creatinine quantification 4
Figure S1. 4
Figure S2. 5
Urinary protein quantification 5
i) Clinical measurements 5
ii) NMR method 5
Figure S3. 6
Statistical analysis 6
i) Probabilistic quotient normalisation 6
Figure S4. 7
ii) Comparisons between PQN coefficients, protein, and creatinine 7
Figure S5 8
iii) Comparison between CPMG and SMolESY estimated PQN coefficients in human plasma heparin ^{1}H NMR spectra. 8
Figure S6 9
References 10

Urine Samples collection

Patients were recruited using the ISARIC WHO Clinical Characterisation Protocol for Severe Emerging Infections in the UK (study registration ISRCTN 66726260). Up to 10 ml of urine biofluid was collected in a sterile universal container and stored at -80°C on site prior to shipment in dry ice to the analytical laboratory (National Phenome Centre at Imperial College London). Ethical approval was provided by the South Central - Oxford C Research Ethics Committee in England (Ref 13/SC/0149), the Scotland A Research Ethics Committee (Ref 20/SS/0028), and the WHO Ethics Review Committee (RPC571 and RPC572, 25 April 2013).

NMR samples preparation and spectra acquisition

The general procedure of NMR samples preparation is described in detail in Dona et al.[1] Briefly, NMR samples were prepared into 96-well plates by adding 630 µL of urine sample to each well and mixing with 70 µL of urine buffer [urine buffer: 1.5 M KH_2PO_4 dissolved in 99.9% ^2H_2O, pH 7.4, 2 mM NaN_3 and 5.8 mM 3-(trimethyl-silyl)propionic acid-d4 (TSP)]. For each sample, 600 µL of the mixture was transferred into a 5 mm NMR tube of the Sample-Jet. Solution ^1H NMR spectra of all samples were acquired using a Bruker IVDr 600 MHz spectrometer (Bruker BioSpin) operating at 14.1 T and equipped with a 5 mm PATXI H/C/N with ^2H-decoupling probe including a z-axis gradient coil, an automatic tuning-matching (ATM) and an automatic refrigerated sample changer (Sample-Jet). Temperature was regulated to 300 ± 0.1 K. For each urine NMR sample, two NMR experiments were acquired in automation: a general profile ^1H NMR water presaturation experiment using a one-dimensional nuclear Overhauser enhancement spectroscopy (1D-NOESY) pulse sequence (where the mixing time of the experiment is used to introduce a second presaturation time) and a 2D J-resolved experiment. Free induction decays of all 1D-spectra were multiplied by an exponential function equivalent to 0.3 Hz line-broadening before applying Fourier transform. All Fourier transformed spectra were automatically corrected for phase and baseline distortions and referenced to the TSP singlet at 0 ppm. For quality control (QC) assessment a pooled QC sample was generated by combining equal parts of each study sample, pooled QC samples prepared as above and spectra acquired regularly throughout the sample analysis.

SMolESY data preparation – Creatinine quantification

SMolESY processing of the standard 1D ^{1}H NMR spectra was performed using the SMolESY platform (https://github.com/pantakis/SMolESY_platform)[2] by employing the fid and the processed dispersive spectral part of the 1D ^{1}H NMR spectra. Protein broad signals were suppressed by SMolESY and sharp signals of small molecules were instantly deconvoluted from both overlapping signals of other metabolites and protein baseline (Figure S1).

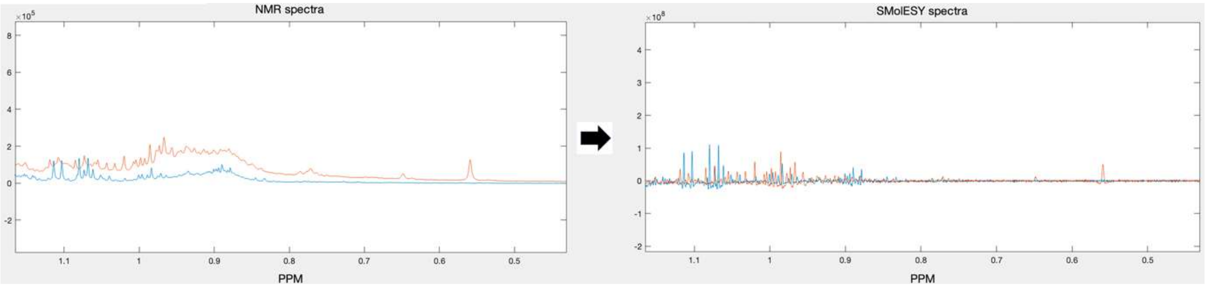

Figure S1. Comparison of two urine ^{1}H NMR spectra (left panel) versus their corresponding SMolESY data (right panel). Both panels are taken from SMolESY platform focusing on the 0.45 – 1.15 ppm, where the baseline elevation due to urinary protein concertation increase is observed in the orange spectrum. Additionally, the SMolESY effectiveness on macromolecular broad signals /baseline suppression.

Consequently, we employed the SMolESY data and the ERETIC signal (i.e. Bruker IVDr) as a reference to absolutely quantify urinary creatinine in all spectra (Figure S2a). To validate NMR quantification, creatinine concentration was measured in a subset of 50 urine samples by a gold-standard clinical method (kinetic alkaline picrate). Clinical creatinine measurements were obtained at North West London Pathology using a standard reagent kit (07P99 Alinity c Creatinine Reagent Kit) with a wavelength setting of 500nm on an Abbott Architect c-8000 platform analyzer (Maidenhead, UK). Comparisons between clinical and NMR-derived

creatinine measurements show excellent linear correlation ($R^2 = 0.96$) (Figure S2b).

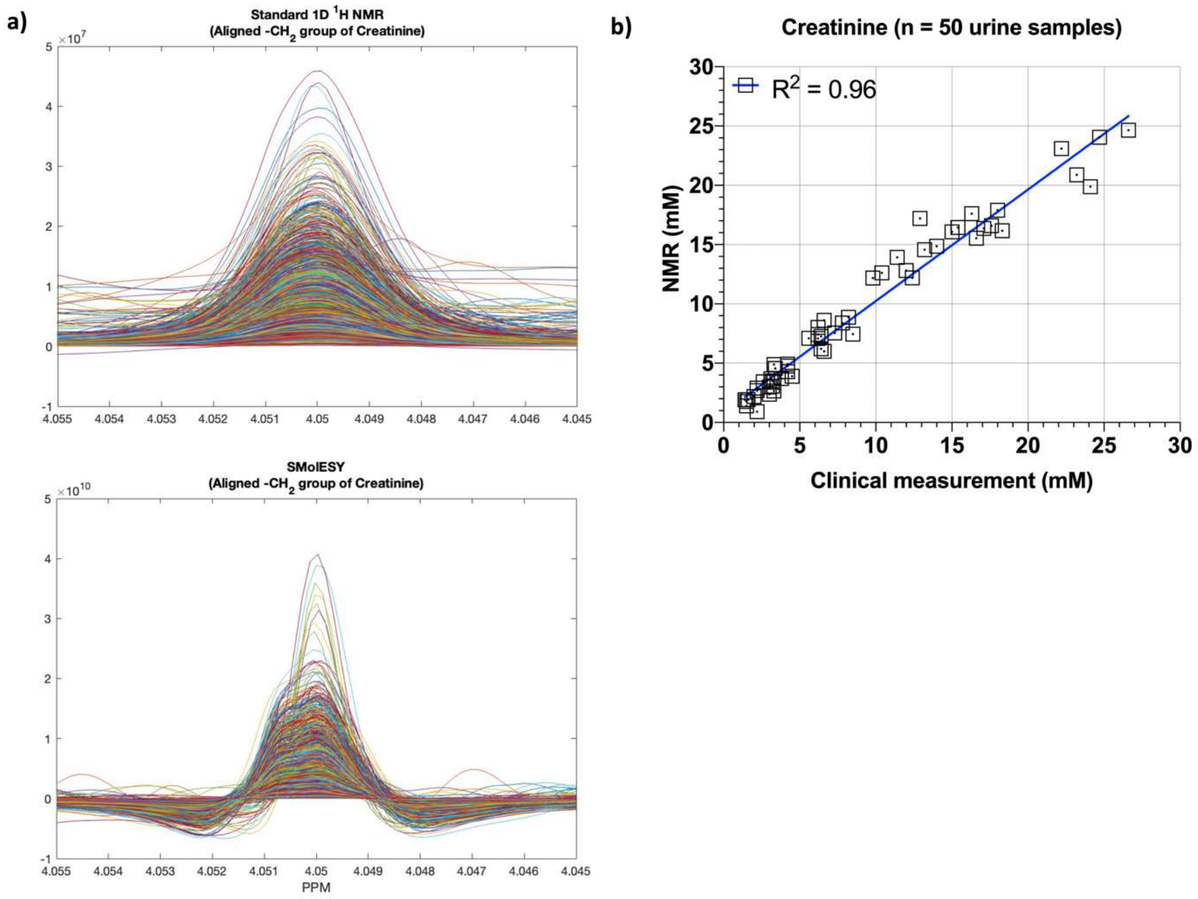

Figure S2. a) The assigned/aligned -CH_2 ^{1}H NMR singlet from creatinine in all urine NMR spectra (top panel) and corresponding SMolESY data (bottom panel), as employed in our study. SMolESY clearly suppresses baseline contributions from proteinic content and thus provides improved concentration measurement. b) Clinical creatinine measurement validates SMolESY NMR creatinine quantification ($R^2 = 0.96$) in a subset of 50 samples.

Urinary protein quantification

i) Clinical measurements

Total urine protein was quantified turbidimetrically on the Abbott Architect c-8000 platform (Maidenhead, UK) using a standard reagent kit (07P59 Alinity c Urine/CSF Protein Reagent Kit) and a wavelength setting of 404 nm.

ii) NMR method

To quantify the total protein via the urine ^{1}H NMR spectra, the recently published method of Vuckovic et al.[3] was followed. In particular, the modified 'reverse' SMolESY for each spectrum was subtracted from its corresponding standard 1D ^{1}H NMR spectrum, that provides the

elimination of all sharp signals from small metabolites, resulting into the ^{1}H NMR spectrum baseline. The spectral region of 0.2– 0.5 ppm (i.e. representing part of the methyl total protein protons) was integrated and ERETIC signal was used for absolute quantification after applying the proposed calibration factors of the above-mentioned published research. Figure S3 shows the application of the described method in one urine spectrum of our study.

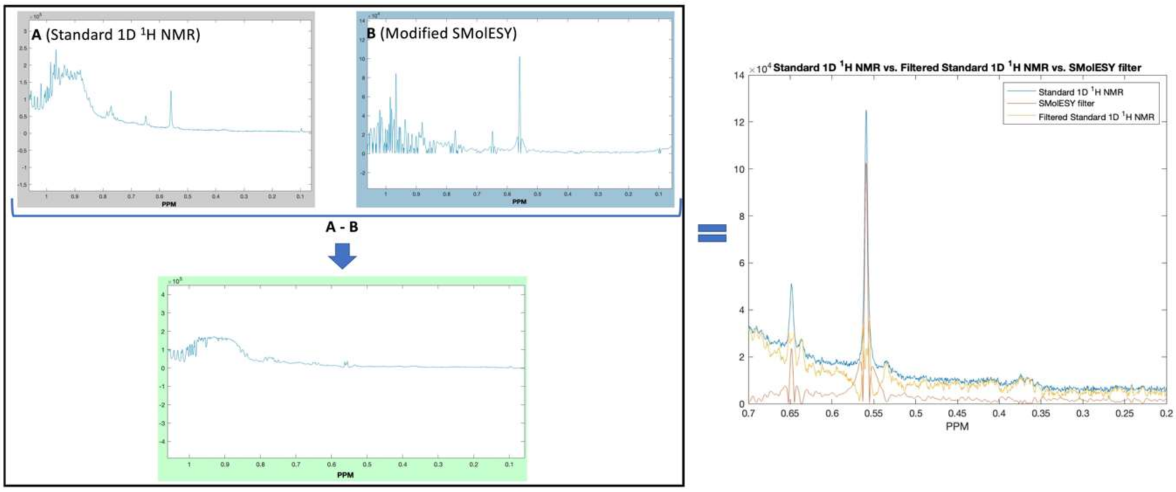

Figure S3. (left panel) By subtracting modified SMolESY from the ^{1}H NMR spectrum, the resulting baseline reflects into the total protein of the urine sample and the maximum number of sharp signals of small metabolites are either eliminated or appear with negative-positive parts which provide ~0 integrals. (right panel) The integration of the 0.2–0.5 ppm region of the filtered from small molecules ^{1}H NMR spectrum (yellow line) provides a good estimation of the total urinary protein.

Statistical analysis

i) Probabilistic quotient normalisation

Probabilistic quotient normalisation was calculated as shown in Dieterle et al[4], but without an initial total area (TA) normalisation step. In TA normalisation, each variable is divided by the row-wise sum (total area) of all variables. Although TA normalisation was recommended as the first step in the original PQN algorithm to "scale individual studies to the same absolute magnitude", we do not consider this step necessary. Moreover, total area normalisation renders the data compositional, breaking the relative quantitative relationship between signal intensity values and compound concentrations, which is necessary to accurately estimate the sample dilution factors. We have assessed the impact of TA normalisation through comparison of the PQN coefficients estimated from the standard non-SMolESY processed NMR spectra with and without TA with urinary creatinine and osmolality measured by the freezing point depression method (using an Advanced® Model 3320 osmometer (MA,

USA)). Prior TA normalisation resulted in PQN coefficients with very poor correlation with creatinine concentration and osmolality (Figure S4). Therefore, all usages of PQN normalisation described in the main text omitted this step. The median spectrum of each dataset was selected as the normalisation reference.

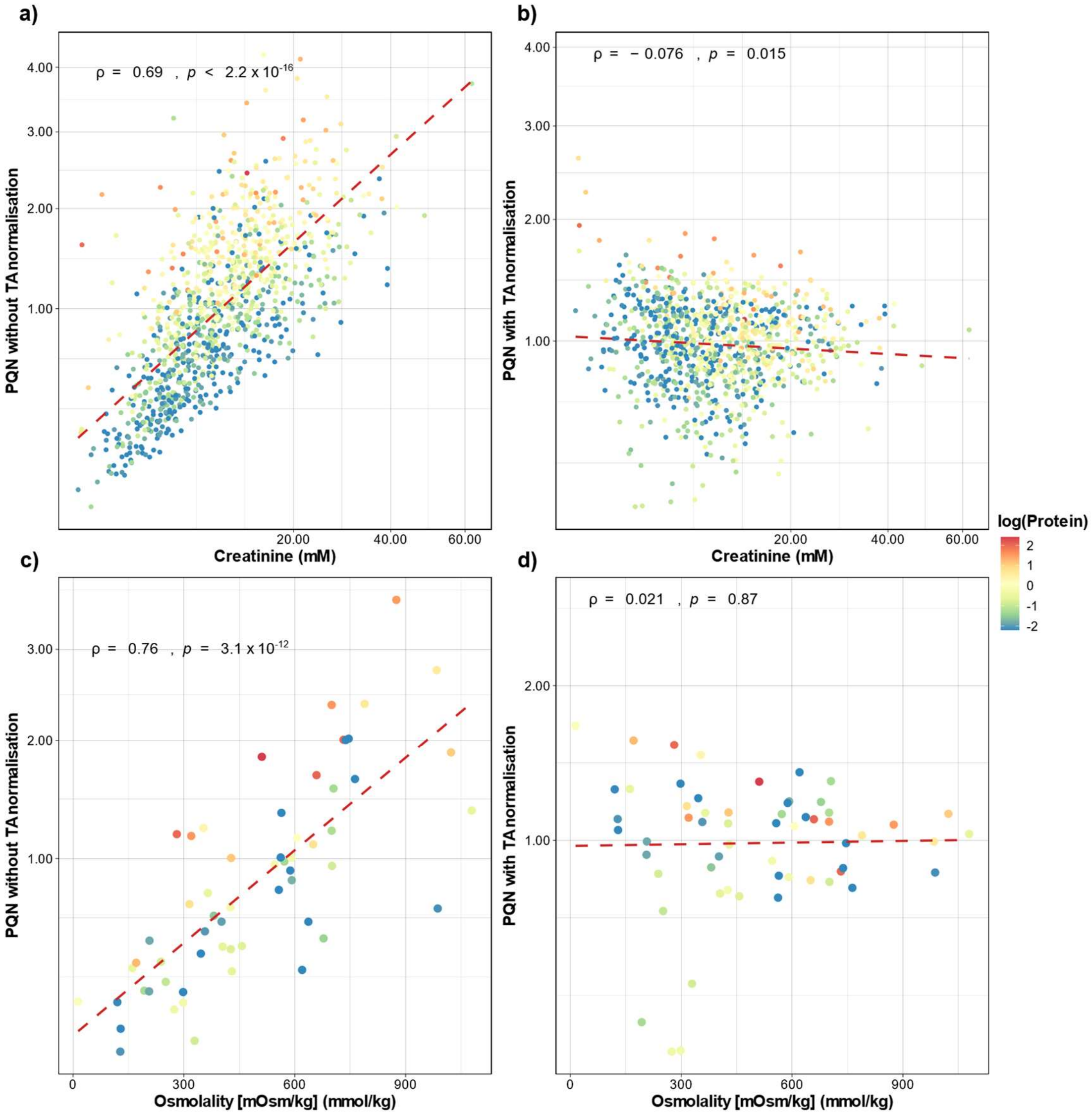

Figure S4. Comparison of PQN coefficients estimated without (A and C) and with (B and D) prior total area normalisation. The correlation between the estimated PQN coefficients and creatinine (A and B) or osmolality (C and D) is absent when the total area normalisation step is performed (B and D). Creatinine and total protein concentrations were square root and log-transformed, respectively. The Pearson correlation coefficient (ρ), the p-value (p) from the two-sided significance test of the correlation coefficient, and the estimated linear regression trendline (dashed red line) are shown in each scatterplot.

ii) Comparisons between PQN coefficients, protein, and creatinine.

Total protein concentration values below the limit of detection (LOD) value of 0.11 mg/ml were imputed by replacement with the LOD, except otherwise stated. Figures 3, 4, 5, 6, S4, S5, and S6 were generated in R (v4.0.3), using the packages 'ggplot2' (v3.3.5), 'ggsignif'

(v0.6.3), and 'gridExtra' (v2.3), 'deming' (v1.4), and 'pcaMethods' (v1.80.0). The Passing-Bablok regression used to compare both PQN coefficients in Figure 3 was fitted with the '*pbreg*' function from the 'deming' with default parameters. Protein concentrations were log transformed, while PQN coefficients and creatinine values were square root transformed, to symmetrize the distribution and account for non-normality and heteroskedasticity.

We have investigated the relationship between urinary creatinine and protein excretion. Division by urinary creatinine concentration is a "gold standard" for spot urine normalisation in clinical assays. We observed a small correlation (ρ = 0.25, Figure S5) between urinary creatinine and protein, without any discernible trend.

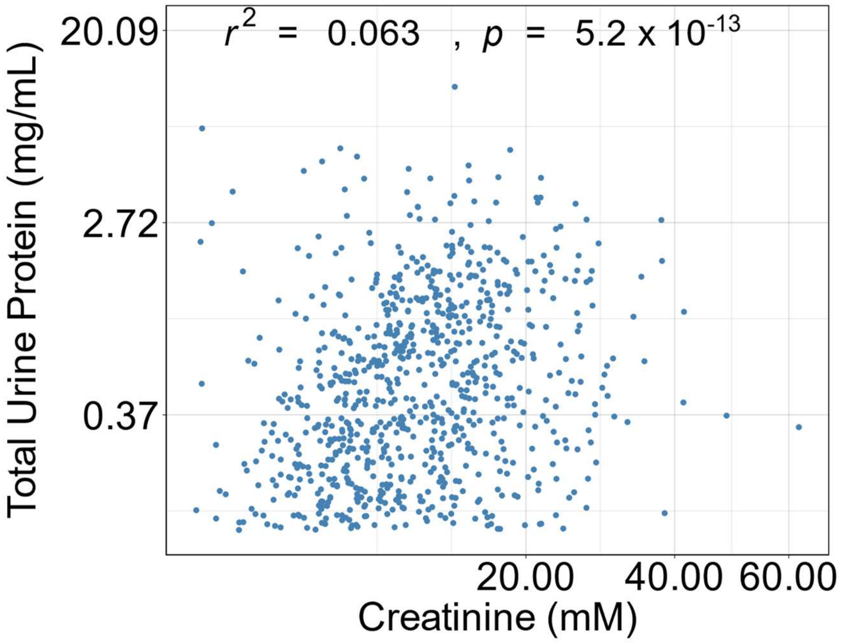

Figure S5. Correlation between urinary creatinine concentration and protein excretion. Creatinine and total protein concentrations were square root and log-transformed, respectively. The Pearson correlation coefficient (ρ) and the p-value (p) from the two-sided significance test of the correlation coefficient are reported. Protein values equal or below the LOD = 0.11 mg/ml were excluded (final n=810).

iii) Comparison between CPMG and SMolESY estimated PQN coefficients in human plasma heparin ^{1}H NMR spectra.

As further evidence that protein baseline signals impact upon the estimation of PQN coefficients, and to compare the performance of SMolESY with an experimental baseline removal procedure, the CPMG pulse sequence, we investigated the relationship between quantified total protein and estimated PQN coefficients in a set (n=322) of ^{1}H NMR spectra from human heparin plasma samples. Figure S6a shows that in a standard 1D pulse sequence

the estimated coefficients have a very high correlation (ρ = 0.9, S6a) with protein concentration. This correlation is greatly reduced in both the CPMG (ρ = 0.17, S6b) and in the SMolESY processed spectra (ρ = 0.19, S6c). CPMG and SMolESY derived PQN coefficients are better correlated (ρ = 0.71, S6f) than with those estimated from the standard 1D spectra ($\rho \leq$ 0.4, S6d and e).

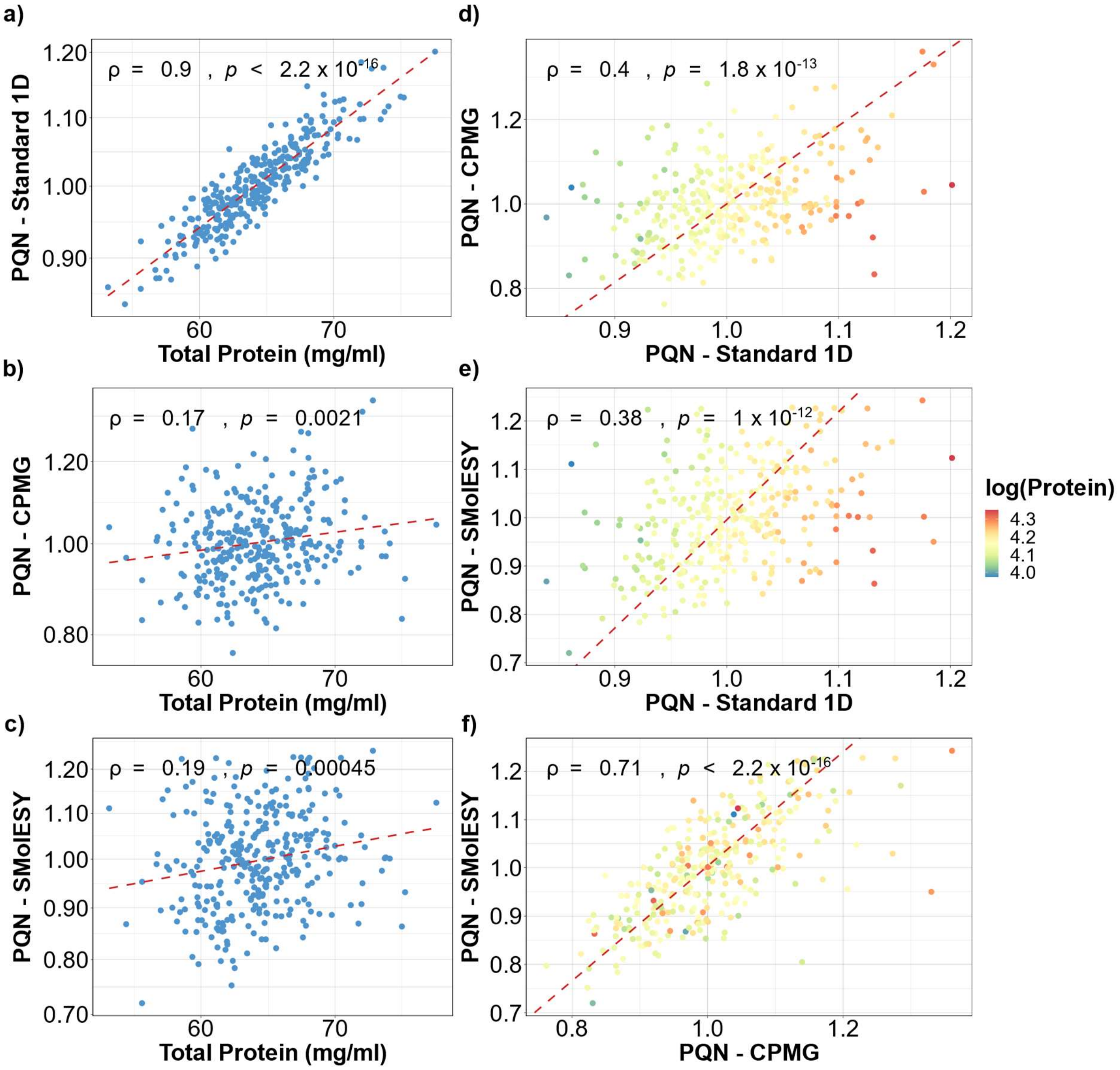

Figure S6. Correlation between total protein and estimated PQN coefficients from standard 1D, CPMG and SMolESY processed spectra in a set 322 human plasma heparin samples. PQN coefficients were estimated from the standard 1D noesy presat (a), CPMG (b), and SMolESY processed spectra (c) and correlated with total plasma protein. The agreement between each set of PQN coefficients is also shown in scatterplots d-f. Total protein concentrations in d-f were log-transformed. The Pearson correlation coefficient (ρ) and the p-value (p) from the two-sided significance test of the correlation coefficient are reported. The linear regression trendline (dashed red line) was estimated with ordinary least-squares in a-c and with the orthogonal least squares Passing-Bablok method in d-f.

The human plasma heparin samples used in these analyses were a random subset (n=322) of ^{1}H NMR 1D NOESY and CPMG spectra taken from the Genetic Regulation of Arterial Pressure

in Humans in the Community (GRAPHIC) study dataset. The GRAPHIC study was approved by the Leicestershire Research Ethics Committee (6463) and all subjects provided written informed consent. The ^{1}H NMR spectra in this dataset were acquired following the protocols for human blood products NMR profiling described in Dona et al[1].

References

(1) Dona, A. C.; Jiménez, B.; Schafer, H.; Humpfer, E.; Spraul, M.; Lewis, M. R.; Pearce, J. T. M.; Holmes, E.; Lindon, J. C.; Nicholson, J. K. Precision High-Throughput Proton NMR Spectroscopy of Human Urine, Serum, and Plasma for Large-Scale Metabolic Phenotyping. *Anal. Chem.* **2014**, *86* (19), 9887–9894. https://doi.org/10.1021/ac5025039.

(2) Takis, P. G.; Jiménez, B.; Sands, C. J.; Chekmeneva, E.; Lewis, M. R. SMolESY: An Efficient and Quantitative Alternative to on-Instrument Macromolecular 1H-NMR Signal Suppression. *Chem. Sci.* **2020**, *11* (23), 6000–6011. https://doi.org/10.1039/D0SC01421D.

(3) Vuckovic, I.; Denic, A.; Charlesworth, M. C.; Šuvakov, M.; Bobart, S.; Lieske, J. C.; Fervenza, F. C.; MacUra, S. 1H Nuclear Magnetic Resonance Spectroscopy-Based Methods for the Quantification of Proteins in Urine. *Anal. Chem.* **2021**, *24*, 27. https://doi.org/10.1021/ACS.ANALCHEM.1C01618/SUPPL_FILE/AC1C01618_SI_003.XLSX.

(4) Dieterle, F.; Ross, A.; Schlotterbeck, G.; Senn, H. Probabilistic Quotient Normalization as Robust Method to Account for Dilution of Complex Biological Mixtures. Application In1H NMR Metabonomics. *Anal. Chem.* **2006**, *78* (13), 4281–4290. https://doi.org/10.1021/ac051632c.
